# Field comparison of circulating antibody assays *versus* circulating antigen assays for the detection of schistosomiasis japonica in endemic areas of China

**DOI:** 10.1186/1756-3305-7-138

**Published:** 2014-03-31

**Authors:** Yu-Chun Cai, Jun-Fang Xu, Peter Steinmann, Shao-Hong Chen, Yan-Hong Chu, Li-Guang Tian, Mu-Xin Chen, Hao Li, Yan Lu, Ling-Ling Zhang, Yang Zhou, Jia-Xu Chen

**Affiliations:** 1 National Institute of Parasitic Diseases, Chinese Center for Disease Control and Prevention, Shanghai 200025, People’s Republic of China; 2 Key Laboratory of Parasite and Vector Biology, Ministry of Health, Shanghai 200025, People’s Republic of China; 3 WHO Collaborating Centre for Malaria, Schistosomiasis and Filariasis, Shanghai 200025, People’s Republic of China; 4 School of Medicine, Hubei University for Nationalities, Enshi, Hubei 445000, P.R. China; 5 Department of Epidemiology and Public Health, Swiss Tropical and Public Health Institute, 4051 Basel, Switzerland; 6 University of Basel, 4051 Basel, Switzerland

**Keywords:** *Schistosoma japonicum*, Circulating antibody, Circulating antigen, China

## Abstract

**Background:**

Schistosomiasis remains a serious public health problem in affected countries, and routine, highly sensitive and cost-effective diagnostic methods are lacking. We evaluated two immunodiagnostic techniques for the detection of *Schistosoma japonicum* infections: circulating antibody and circulating antigen assays.

**Methods:**

A total of 1864 individuals (between 6 and 72 years old) residing in five administrative villages in Hubei province were screened by serum examination with an indirect hemagglutination assay (IHA). The positive individuals (titer ≥20 in IHA) were reconfirmed by stool examination with the Kato-Katz method (three slides from a single stool specimen). Samples of good serum quality and a volume above 0.5 ml were selected for further testing with two immunodiagnostic antibody (DDIA and ELISA) and two antigen (ELISA) assays.

**Results:**

The average antibody positive rate in the five villages was 12.7%, while the average parasitological prevalence was 1.50%; 25 of the 28 egg-positive samples were also circulating antigen-positive. Significant differences were observed between the prevalence according to the Kato-Katz method and all three immunodiagnostic antibody assays (P-value <0.0001). Similar differences were observed between the Kato-Katz method and the two immunodiagnostic antigen assays (P-value <0.0001) and between the antigen and antibody assays (P-value <0.0001).

**Conclusion:**

Both circulating antibody and circulating antigen assays had acceptable performance characteristics. Immunodiagnostic techniques to detect circulating antigens have potential to be deployed for schistosomiasis japonica screening in the endemic areas.

## Background

Schistosomiasis remains a serious public health problem in endemic countries [1-3], including in the People’s Republic of China. According to the national annual disease report, there were 365,770 *Schistosoma japonicum* patients and approximately 250 million people at risk of infection in China in 2009 [4]. After a half-century fight against schistosomiasis japonica, the prevalence and intensity of *S. japonicum* infection have decreased significantly [5-8]. Today, the prevalence is relatively low in most of the traditional endemic areas. Therefore, cost-effective routine methods for diagnosis are now required for deployment in low-endemic areas and for the correct diagnosis of infections in travelers and migrants [9-11]. Currently available diagnostic methods include direct parasitological (parasite egg detection and miracidium hatching), direct serological (circulating antigens) and indirect serological techniques (circulating antibodies). Microscopic examination of stool is traditionally considered the “gold standard” for the diagnosis of schistosomiasis [12]. However, the involved procedures (mainly Kato-Katz thick smears and hatching in water) are time-consuming and have limited sensitivity due to the day-to-day fluctuations in egg output [13,14]. It must also be considered that for the Kato-Katz method, only 41.7 mg of fecal material are examined per slide, limiting the chance to detect eggs in the case of light-intensity infections. This results in high rates of false-negative results in certain populations, most notably following repeated rounds of mass praziquantel administration, the current mainstay of schistosomiasis control worldwide [15-19]. It follows that the current diagnostic gold standard may be unsuitable for surveys and surveillance in communities with predominantly low-intensity infections [20-25].

Immunodiagnostic techniques may be used to detect circulating antigens of schistosomes and to detect host antibodies against the parasite. However, the immunologic diagnosis is usually not species-specific and may not reliably indicate cure in the short term [26,27]. Studies showed that the false-positive rates of the indirect hemagglutination test (IHA) and enzyme linked immunosorbent assays (ELISA) were very high in field settings where such tests were used to identify villagers infected with *S. japonicum*[28,29]. Theoretically, the false positives might be stratified into three groups: 1) infected individuals who were misdiagnosed by stool examination; 2) previously infected individuals who were cured following treatment; 3) cross-reactions between antigens of/antibodies to other parasites than *S. japonicum*. In addition, it has been observed that immunodiagnostic assays also missed a certain number of light-intensity infections, especially in areas of relatively severe endemicity [30].

Okabe & Tanaka [31] arguably were the first ones who described that antigens secreted by parasites and circulating in the host blood might be a potential diagnostic material [32,33]. Circulating anodic antigen (CAA) and circulating cathodic antigen (CCA) are adult worm gut-associated antigens. Serum levels of CAA are related to actual worm burden and rapidly decrease following drug treatment [34-38]. Soluble egg antigens (SEA) of *S. japonicum* represent schistosome egg metabolic and secreted antigens in a patient’s blood, urine or other body fluids. Detection of SEA thus is a more direct measure of the presence of worms, providing accurate information on the status and intensity of infection. Although the schistosome species currently cannot be determined based on secreted antigens, the detection of circulating antigens (SEA, CCA and CAA) has good specificity for the diagnosis of *Schistosoma* spp infections, and there are very few false-positive diagnoses with these methods [39,40].

## Methods

### Ethics statement

The study was authorized by the ethics committee of IPD, China CDC (Ref No: 20100802–1). All participants were informed about the study aims and procedures, following which they gave written informed consent to participate. Participants who were found to be parasitologically positive were given free praziquantel treatment.

### Study population

A total of 1864 individuals residing in five administrative villages in Jiangling county, Hubei province participated in this study. All participants were aged between 6 and 72 years at the time of the study (August and September 2010). All participants were screened by serum examination with an indirect hemagglutination assay (IHA, LOT: 20100608 from Anji Pharmaceutical Science and Technology Co. Ltd.). Infections were confirmed by stool examination with the Kato-Katz technique for individuals with titers ≥20. Each participant was asked to provide one blood sample of about 2–3 ml collected by venipuncture, and positive individuals were asked to provide one stool sample of over 50 g. The samples were transferred to the local schistosomiasis stations where IHA and Kato-Katz tests were performed by laboratory staff within 24 hours of sample collection. All serum samples of good quality and a volume above 0.5 ml were selected for further testing. Immunodiagnostic antibody (DDIA and ELISA) and antigen (ELISA) assays were performed by trained staff of the National Institute of Parasitic Diseases, Chinese Center for Disease Control and Prevention (IPD, China CDC) in Shanghai after the field work.

### Stool examinations

The Kato-Katz thick smear method was used to detect schistosome eggs in the collected stool samples. Three slides were prepared per sample, using a standard template holding 41.7 mg. Each slide was checked by two experienced technicians 12–48 h after their initial preparation. Eggs per gram of stool (EPG) was used to express the infection intensity, calculated as the arithmetic mean of the egg counts obtained from the three slides multiplied by 24 [41].

### Immunodiagnostic antibody assays – IHA, ELISA and DDIA

#### Indirect hemagglutination assay (IHA)

The IHA test kits were purchased form the Anhui provincial Institute of Parasitic Diseases, P.R China. The test instructions were accurately followed and all sera were tested by this method. In brief, 100 μl of normal saline solution was added to the first well of the plate in the transverse line and 25 μl of normal saline solution were added to the wells 2 and 3. A total of 25 μl serum was then added to the first well and thoroughly mixed with the saline solution. In the next step, 25 μL of the mixture in the first well was transferred to the second well and mixed as before. The same procedure was repeated for the third well. Hence, the concentration of the second well was 1:10 and that of the third well was 1:20. Positive and negative control sera were tested simultaneously on each Plate. 25 μl of 2.5% sensitized red blood cells was placed into each well, shaken, and kept at 37°C for half an hour. The results of the test were read by eye. The terminal point of a positive reaction was the highest titer where agglutination appeared. If a positive reaction appeared at a titer 1:10, the sample was considered positive [42].

#### ELISA antibody assay

The ELISA test kits for detecting anti-schistosome antibodies were purchased form Shenzhen Combined Biotech Co., P.R China [43]. The experiments were operated according to the manufacturer’s instructions. To each well of the ELISA plate were added 100 μl of the patient serum (1:100 diluted), and the plate was incubated at 37°C for half an hour. The plate was then washed 3 times with washing buffer. 100 μl of a peroxidase-conjugated goat anti-human IgG antibody was added, and the plate was incubated again at 37°C for half an hour. After that the plate was washed, tetramethyl benzidine (TMB) substrate was added to each well, and the plate was incubated for 10 minutes at 37°C. The reaction was stopped by adding 50 μl of 2 m sulfuric acid to each well. The optical density (O.D.) was measured at 450 nm.

#### Dipstick Dye Immunoassay (DDIA)

The serum samples were also tested by the DDIA method [5], with DDIA kits purchased form Wuxi Saide Medical Technology Co. Ltd., P.R. China. The tests were performed according to the instructions supplied by the manufacturer. In brief, to each polyvinyl chloride (PVC) well a drop of blue colloidal dye-labeled soluble egg antigens (SEA) solution was added, followed by 20 μl of serum. The content of the well was then mixed gently for one minute, and a dipstick was inserted into the well. Ten minutes later, when the solution was absorbed completely, the result was read by eye. Positive samples had two blue bands on the control line and the test line of the dipstick, while a negative one only had one single blue band on the control line.

#### ELISA antigen assay

Two ELISA kits were used to detecting circulating antigens in all serum samples. One was purchased from Sichuan Maker Biotechnology Co. P.R. China (which was designed to detecting circulating *S. japonicum* SEA antigen in the serum of patients). The second one was a sandwich ELISA established by our laboratory using a combination of anti-*S. japonicum* SEA-IgY polyclonal antibodies and anti-*S. japonicum* SEA-IgM monoclonal antibodies. The specificity of the sandwich ELISA for detecting circulating *S. japonicum* SEA antigen in the serum of infected individuals has been established [44]. The steps involved in testing a sample with the two kits were basically the same as described for the ELISA antibody assay. The main difference was that each well of the commercial ELISA was loaded with 50 μl serum directly, while the ELISA developed by our lab required 100 μl serum solution in each well (1:5 diluted).

### Statistical analysis

Groups were compared by the Chi-square test or Fisher’s exact test as appropriate. Differences were considered significant when the P-value was less than 0.05. 95% confidence intervals (CIs) to the prevalence values of each method were also calculated.

## Results

### The sero-prevalence of *Schistosoma japonicum* according to different methods

Among the 1864 individual sera, 240 were antibody positive as tested with the IHA method in the field immediately after the serum samples were collected. This translates into an average antibody positive rate across the five villages of 12.7%. The ELISA method gave the same results as the IHA but the number of positive samples according to the DDIA method was a little smaller than with the two other methods. When tested for antigens produced by the parasite, average positive rates of respectively 4.7% (ELISA (COM)) and 4.3% (ELISA (IgY)) were recorded. Among the 240 fecal samples from IHA positive participants, *S. japonicum* eggs were detected in 28 with the Kato-Katz method. Thus, the average parasitological prevalence in the five villages was 1.50% (Table 1).

**Table 1:** **Prevalence of *Schistosoma japonicum *** **according to different diagnostic methods**

**Village**	**Total**	**Kato-Katz method**		**Immunodiagnostic antibody assay**					**Immunodiagnostic antigen assay**			
		**Positive (number)**	**Positive (%) [95% CI]**	**IHA**	**ELISA**	**DDIA**	**Average (number)**	**Average (%) [95% CI]**	**ELISA (COM)**	**ELISA (IgY)**	**Average (number)**	**Average (%) [95% CI]**
Village A	544	1	0.2 [0.03-0.33]	30	30	29	29.7	5.5 [3.58-7.42]	15	14	14.5	2.7 [1.34-4.06]
Village B	239	1	0.4 [0.08-0.75]	11	11	11	11	4.6 [1.94-7.26]	1	1	1	0.4 [0.08-0.75]
Village C	311	7	2.3 [0.63-4.0]	44	44	44	44	14.1 [10.23-17.97]	18	15	16.5	5.3 [2.81-7.79]
Village D	361	10	2.8 [1.1-4.5]	87	87	81	85	23.5 [19.13-27.87]	34	30	32	8.9 [5.96-11.84]
Village E	409	9	2.2 [0.78-3.62]	68	68	66	67.3	16.5 [12.90-20.10]	19	21	20	4.9 [2.81-6.99]
Total	1864	28	1.5 [0.95-2.05]	240	240	231	237	12.7 [11.19-14.21]	87	81	84	4.5 [3.56-5.44]

### Major features and statistical comparison of the six different diagnostic methods

**Figure 1:**
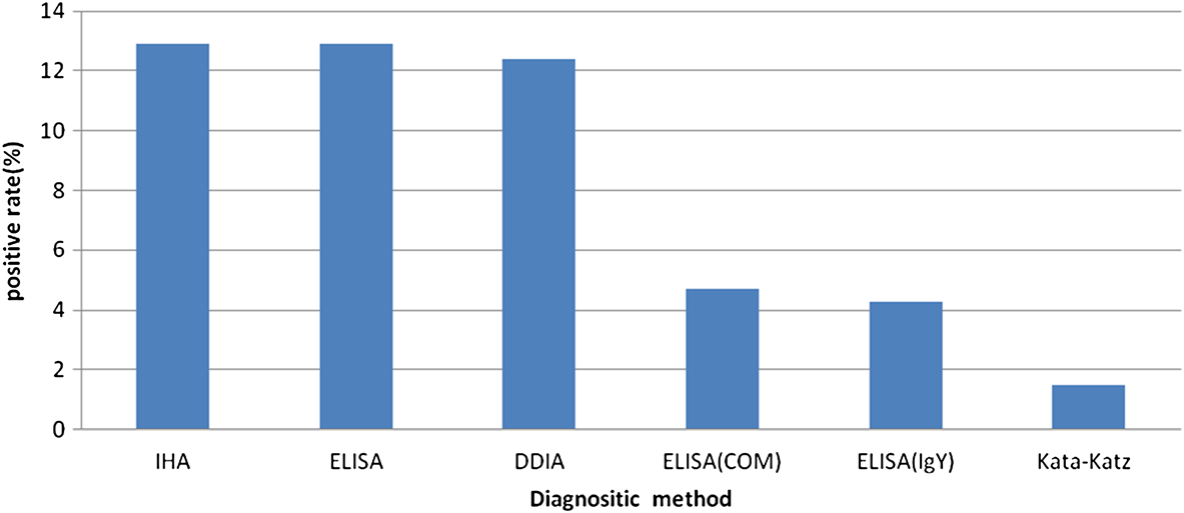
***S. Japonicum *** **prevalence according to six different test systems.**

Major features of the diagnostic tests are listed in Table 2. The three immunodiagnostic antibody assays (IHA, DDIA and ELISA) were all developed in P.R. China and met the national criteria for field use. The antigen used for all tests was a crude soluble *S. japonicum* egg extract and the specimen tested in all kits was serum. However, the sample volume differed between assays: an ELISA test needs just 1 μl serum whereas the IHA assay and the DDIA assay need 25 μl and 10 μl, respectively. The two immunodiagnostic antigen assays were both ELISA tests. The Kato-Katz method was the only parasitological method we used in this study. Significant differences were observed between the prevalence according to the Kato-Katz method and all the five immunodiagnostic assays (Figure 1 and Table 3).

**Table 2:** Key characteristics of six different diagnostic methods

**Assay**	**Tested samples**	**Positive samples**	**Adjusted prevalence (%) [95% CI]**	**Detection target**	**Solid phase**	**Time required per sample**	**Volume of sample**	Extra supplies*
IHA	1864	240	12.9 [11.38-14.42]	Anti-SEA antibody	Red blood cells from sheep	40 min	25 μl	Yes
ELISA	1864	240	12.9 [11.38-14.42]	Anti-SEA antibody	Microtitre plate	90 min	1 μl	Yes
DDIA	1864	231	12.4 [10.90-14.90]	Anti-SEA antibody	Nitrocellulose membrane	15 min	10 μl	No
ELISA (COM)	1864	87	4.7 [3.74-5.66]	Circulating antigen	Microtitre plate	90 min	50 μl	Yes
ELISA (IgY)	1864	81	4.3 [3.40-5.22]	Circulating antigen	Microtitre plate	90 min	20 μl	Yes
Kato-Katz	1864	28	1.5	Egg	Plastic template	40 min	41.7 mg	Yes

*Additional equipment required for all serological tests consisted of handheld micropipettes and micropipette tips.

**Table 3:** **Statistical comparison of the results from six different methods to diagnose *S. japonicum *** **infections (P values)**

**Type**		**Circulating antibody assays**			**Circulating antigen assays**		**Parasitological method**
		**IHA**	**ELISA**	**DDIA**	**ELISA-COM**	**ELISA-IgY**	**Kato-Katz**
Circulating antibody assays	IHA						
	ELISA	1					
	DDIA	0.0495*	0.0495*				
Circulating antigen assays	ELISA-COM	<0.0001**	<0.0001**	<0.0001**			
	ELISA-IgY	<0.0001**	<0.0001**	<0.0001**	0.0833		
Parasitological method	Kato-Katz	<0.0001**	<0.0001**	<0.0001**	<0.0001**	<0.0001**	

*indicates significant difference (p < 0.05);**indicates highly significant difference (p < 0.001).

### Detection of antibodies or antigen in *S. japonicum* egg-positive patients

The 28 *S. japonicum* egg-positive individuals include 7 (25%) women and 21 (75%) men. The median age was 54 years, with a range of 30 to 72 years. Twenty (71.4%) of the egg positive individuals were farmers, and 8 (28.6%) individuals had other occupations. The EPG values ranged between 8 and 736, with 10.7% having ≥100 EPG, 21.4% having 50–100 EPG and 67.9% having ≤50 EPG. No co-infection with other helminths was detected in the 28 samples. The rate of positive results for each immunological antibody and antigen test of the 28 parasitological positive samples is detailed in Table 4. The three antibody tests correctly diagnosed 100.0% of the egg-positive individuals while the two antigen tests detected 93.0% (ELISA (COM)) and 86% (ELISA (IgY)) of the cases. However, this difference was not statistically significant (P > 0.05). The 4 false negative specimens were mostly from patients with EPG values of 24 or less.

**Table 4:** **Detection of antibodies or antigen in *S. japonicum *** **egg-positive patients (n = 28)**

**Assay**	**No. of positives**	**No. of false negatives**	EPG* for misdiagnosed	**Sensitivity (%)**	**P value against Kato-Katz**
IHA	28	0	0	100%	1
ELISA	28	0	0	100%	1
DDIA	28	0	0	100%	1
ELISA(COM)	26	2	8,8	93%	0.48
ELISA(IgY)	24	4	24,8,8,8	86%	0.13

*EPG: egg per gram feces.

## Discussion

The prevention and control of schistosomiasis japonica in P.R. China still faces serious challenges, and accurate diagnosis is crucial for the effective control and surveillance of the disease. According to the latest national epidemiological sampling survey, the average prevalence was 5.1% in the areas where control of schistosomiasis transmission had not yet been achieved, and in all surveyed endemic areas the average prevalence was 2.5% [45]. To achieve the ultimate goal of elimination, a national program was established with the aim of decreasing the prevalence of schistosome infection in all endemic counties below 5% in 2008 and 1% in 2015 [46,47]. The study region in Jiangling county is a hyper-endemic area of *S. japonicum* in accordance with the current national prevalence classification. The five villages can be divided into two different levels, village A and B where the average prevalence is 0.3% according to the gold standard (Kato-Katz method), much lower than in the villages C, D and E where the prevalence was 2.4%.

Serological diagnosis of anti-schistosome antibodies has been widely used in endemic areas because it is more sensitive than parasitological diagnosis and antibodies are easier to detect than antigens [43]. In this study, we used three circulating antibody detection methods, IHA, ELISA and DDIA. They demonstrated a good ability to identify schistosomiasis patients, with positive rates in the range of 12.9%, 12.9% and 12.4%. The IHA and ELISA gave the same results. The antibody ELISA can give quantitative results based on OD values, but the need for a 37°C incubator and a microplate reader to obtain results limits its use. IHA tests are the most widely used assays in P.R. China and only need an incubator. The DDIA method gave the lowest number of positive results but has its own advantages: the time required per sample of DDIA is only 15 min, no extra supplies are needed and it is suitable for large-scale screenings. Despite the advantages of these methods, detection of circulating antibodies also has its disadvantages as detecting antibodies cannot distinguish between current and past infections. Evaluating treatment outcomes is thus impossible, as antibodies are retained in the body for a number of years after treatment [9,48].

Conversely, detection of parasite antigen may indicate the existence of active infection, and can greatly reduce the rate of false-positive results compared to antibody detection tests. This reduces costs of field investigations while improving accuracy of diagnosis. The reduced sample size required for surveillance and improved targeting of infected individuals with this method are particularly welcome as after years and years of praziquantel chemotherapy, treatment compliance in endemic areas is worsening [49,50]. In this study, two antigen detection methods had been evaluated. The prevalence according to the two methods were 4.3% (ELISA (IgY)) and 4.7% (ELISA (COM)). There were no significant differences between the two methods (P = 0.083). The average prevalence according to the two antigen-detecting methods was 4.5%, higher than with the parasitological method (1.5%), and lower than with the antibody-detecting methods (12.7%). The reason probably is that the fraction of truly infected individuals that tested positive in stool examinations was very low [51,52], resulting in many false-negative diagnoses [53-55]. Antigen detection methods may more accurately reflect the existence of active infections in the population, and allow the evaluation of treatment outcomes. However, antigen detection ELISA methods are not a complete solution as we found that among the 28 serum samples from egg-positive individuals, antigens were detected only in 24 samples while 4 samples tested negative (these being subjects with EPG counts of 24 or less). If we accept that stool examination is the “gold standard” for the detection of schistosomiasis and that all individuals with a positive stool examination finding were truly infected with *S. japonicum*, antigen detection would have a sensitivity of 89.3% (25/28). The following points are offered for consideration. 1. There is a problem with false-negative results in ELISA kits for antigen detection; 2. There is no linear relationship between the concentration of circulating antigens in the patient’s body and the number of *S. japonicum* eggs in the patient’s stools as the concentration of circulating antigens is influenced by many other factors, such as the health condition and immune status of the patient, or the formation of circulating immune complexes which are difficult to detect [56].

Both the immunodiagnostic antibody assays (IHA, ELISA and DDIA), and the immunodiagnostic antigen assays (ELISA (IgY) and ELISA (COM)) need collection of whole blood samples via venipuncture. This is a limitation because well trained personnel are required and the practice is not always widely accepted by the population. In recent years, based on the observation that parasite antigens may indicate the existence of an active infection, and its detection can greatly reduce the false-positive rate of antibody detection tests, immunodiagnostic antigen assays (SEA, CCA and CAA) have been heralded as ideal diagnostic tests, and a number of circulating antigen assays have been developed to detect *Schistosoma japonicum* or *Schistosoma mansoni* infections [57-60]. A rapid diagnostic test has also been developed to detect circulating cathodic antigen (CCA) of *Schistosoma mansoni* in urine [61]. In our study we chose to only focus on schistosome egg antigen, and not CCA or CAA. Schistosomiasis japonica is different from the other three schistosome species in that in the chronic stage, patients may develop liver fibrosis, also called schistosome egg-induced fibrosis, indicating that eggs play an important role in the development of the pathology. So though CCA and CAA have the advantage of being antigens directly associated with adult worm feeding, SEA is expected to be more indicative of an active infection than CCA or CAA in the chronic and later stage. The sandwich ELISA developed in our laboratory using a combination of anti-*S. japonicum* SEA-IgY polyclonal antibodies and anti-*S. japonicum* SEA-IgM monoclonal antibodies (ELISA (IgY)) gave almost the same results as the ELISA (COM)) assay which is wildly used in P.R. China. In conclusion, we believe that immunodiagnostic techniques to detect circulating antigens have the potential to be deployed for schistosomiasis japonica screening in the endemic areas.

## Conclusions

Our study confirmed that both circulating antibody and circulating antigen assays had acceptable performance characteristics. But significant differences were observed between the antigen and antibody assays (P-value <0.0001).The sandwich ELISA developed in our laboratory for detecting circulating antigen gave great results in this study, so we believe immunodiagnostic techniques to detect circulating antigens have potential to be deployed for schistosomiasis japonica screening in the endemic areas.

## Competing interests

The authors declare that they have no competing interests.

## Authors’ contributions

Conceived and designed the experiments: YCC, JFX, JXC. Performed the Experiments: YCC, JFX, JG, SHC, MXC, XMT, HL, LGT, LA, LLZ, YZ. Analyzed the data: YCC, JFX, LGT, PS. Wrote the paper: YCC, JFX, JXC, PS. All authors read and approved the final version of the manuscript.
